# Supplementary material for: MRI-derived quantification of hepatic vessel-to-volume ratios in chronic liver disease using a deep learning approach
Source: Eur Radiol Exp. 2025 Aug 12;9:75. doi: 10.1186/s41747-025-00612-y (PMC12343422; doi:10.1186/s41747-025-00612-y)
Supplement: Supplementary file 1 — Supplementary information [file 41747_2025_612_MOESM1_ESM.pdf]

MRI-Derived Quantification of Hepatic Vessel-to-Volume Ratios in Chronic Liver Disease Using a Deep Learning Approach

ELECTRONIC SUPPLEMENTARY MATERIAL

Subgroup Analysis of Hepatic Vessel-to-Volume Ratios:

To further analyze the chronic liver disease group and explore the relationship between progressive liver disease severity and vessel-to-volume ratios, we performed two additional subgroup analyses beyond the main three-group comparison (healthy controls, non-ACLD, and ACLD) presented in the manuscript.

First, we stratified the chronic liver disease patients into three categories: non-ACLD (n=44), compensated ACLD (cACLD, n=74), and decompensated ACLD (dACLD, n=44). This classification aligns with the Baveno VII consensus [21] that defines cACLD as patients with untreated/active chronic liver disease at risk of having clinically significant portal hypertension, and consequently, at risk of decompensation and liver-related death.

As shown in Table S2, when dividing patients into non-ACLD, cACLD, and dACLD groups, we observed significant differences in TVVR (p=0.0017) and HVVR (p<0.0001) across groups, while PVVR remained stable (p=0.4021). Post-hoc pairwise comparisons revealed that TVVR significantly differed between non-ACLD and cACLD (p=0.033) as well as between non-ACLD and dACLD (p=0.001), but not between cACLD and dACLD (p=0.443). Similarly, HVVR showed significant differences between non-ACLD and cACLD (p<0.001) and between non-ACLD and dACLD (p<0.001), but not between cACLD and dACLD (p=1.000). These findings are visually represented in Figure S1.

Second, we stratified patients based on FIB-4 score cutoffs (<1.3, 1.3-2.67, >2.67) representing low, intermediate, and high risk for advanced fibrosis, respectively following the established thresholds as mentioned by Sterling et al. [29].

Similarly, our FIB-4 score-based stratification (Table S3) demonstrated significant differences in TVVR (p=0.0072) and HVVR (p<0.0001) across the three groups, with no significant differences in PVVR (p=0.4730). Pairwise comparisons showed significant differences in TVVR between FIB-4 <1.3 and 1.3-2.67 (p=0.026) and between FIB-4 <1.3 and >2.67 (p=0.010). For HVVR, significant differences were observed between FIB-4 <1.3 and 1.3-2.67 (p=0.001) and between FIB-4 <1.3 and >2.67 (p<0.001). These findings are illustrated in Figure S2.

Supplemental Tables:

Table-S1. MRI protocol

Sequence	Section Thickness (mm)	TR (msec)	TE (msec)	FOV (mm)	Phase Direction	Flip Angle
GRE-T1 (flash 2D) in-phase	5	130	2.5	350	AP	70
GRE-T1 (flash 2D) opp-phase	5	131	3.7	350	AP	70
T1 VIBE SPAIR axial	1.7	2.7	1.0	430	AP	13
T1 VIBE SPAIR coronal	2	2.6	0.9	500	RL	13
T2 Haste coronal	4.5	805	76	450	RL	141
DWI axial TSE-EP	6	1700	73	380	AP	—
T2 MRCP 2D-HASTE	45	5500	454	380	RL	180
T2 MRCP 3D-SPACE	0.9	2400	707	350	RL	variable
T2 Haste axial fs.	5	1800	150	400	AP	150

Table S1: MRI = magnet resonance imaging; 2D = Two-dimensional; FOV = field of view; fs = fat saturation; GRE = gradient echo; SPAIR = spectral attenuated inversion recovery; TE = echo time; TR = repetition time; TSE = turbo spin echo; VIBE = volumetric interpolated breath-hold examination; MRCP = magnetic resonance cholangiopancreatography; HASTE = Half-Fourier-Acquired Single-shot Turbo spin Echo.

Table-S2. Vessel-to-volume ratios by CLD Group

Parameter	non-ACLD (n=44)	cACLD (n=74)	dACLD (n=44)	p-value*	Post-hoc comparisons†
**TVVR**	2.8 (2.3-3.8)	2.3 (1.8-3.3)	2.3 (1.5-3.0)	0.0017	a: p=0.033, b: p=0.001, c: p=0.443
**HVVR**	1.7 (1.1-2.2)	1.0 (0.7-1.5)	0.9 (0.7-1.4)	<0.0001	a: p<0.001, b: p<0.001, c: p=1.000
**PVVR**	1.2 (0.9-1.6)	1.3 (0.9-1.6)	1.20 (0.8-1.6)	0.4021	a: p=1.000, b: p=1.000, c: p=0.584

Table S2: Comparison of vessel-to-volume ratios across clinical disease stages. The Kruskal-Wallis test was used to assess overall differences between groups, followed by pairwise Mann-Whitney U tests with Bonferroni correction for multiple comparisons. *Kruskal-Wallis test; †Post-hoc pairwise comparisons (Dunn's test with Bonferroni correction): a: non-ACLD vs cACLD, b: non-ACLD vs dACLD, c: cACLD vs dACLD. TVVR = total vessel-to-volume ratio; HVVR = hepatic vein-to-volume ratio; PVVR = portal vein-to-volume ratio; non-ACLD = non-advanced chronic liver disease; cACLD = compensated advanced chronic liver disease; dACLD = decompensated advanced chronic liver disease.

Table-S3. Vessel-to-volume ratios by FIB-4-Score

Parameter	FIB4 <1.3 (n=46)	FIB4 1.3-2.67 (n=47)	FIB4 >2.67 (n=69)	p-value*	Post-hoc comparisons†
**TVVR**	2.8 (2.4-3.8)	2.3 (1.7-3.2)	2.3 (1.6-3.0)	0.0072	a: p=0.026, b: p=0.010, c: p=1.000
**HVVR**	1.7 (1.3-2.2)	0.9 (0.7-1.6)	1.0 (0.7-1.4)	<0.0001	a: p=0.001, b: p<0.001, c: p=1.000
**PVVR**	1.2 (0.9-1.7)	1.1 (0.9-1.4)	1.4 (0.9-1.8)	0.4730	a: p=1.000, b: p=1.000, c: p=0.665

Table S3: Comparison of vessel-to-volume ratios stratified by FIB-4 score categories. The Kruskal-Wallis test was used to assess overall differences between groups, followed by pairwise Mann-Whitney U tests with Bonferroni correction for multiple comparisons. Significant p-values are highlighted in bold. *Kruskal-Wallis test; †Post-hoc pairwise comparisons (Dunn's test with Bonferroni correction): a: <1.3 vs 1.3-2.67, b: <1.3 vs >2.67, c: 1.3-2.67 vs >2.67; TVVR = total vessel-to-volume ratio; HVVR = hepatic vein-to-volume ratio; PVVR = portal vein-to-volume ratio.

Supplemental Figure Legends:

Fig. S1:

Fig. S1: Distribution of vessel-to-volume ratios across clinical disease stages. Box plots show total vessel-to-volume ratio (TVVR), hepatic vein-to-volume ratio (HVVR), and portal vein-to-volume ratio (PVVR) across non-advanced chronic liver disease (non-ACLD), compensated advanced chronic liver disease (cACLD), and decompensated advanced chronic liver disease (dACLD) groups.

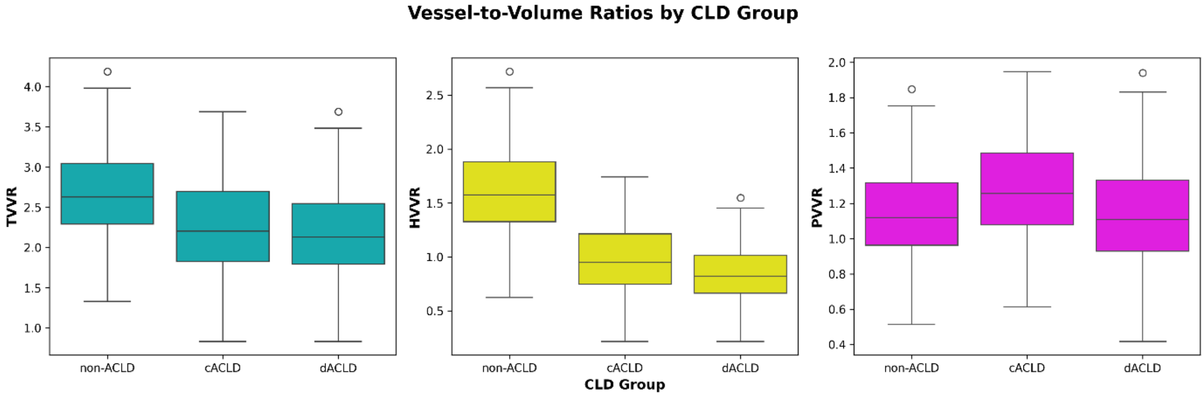

Fig. S2:

Fig. S2: Distribution of vessel-to-volume ratios across FIB-4 score categories. Box plots show total vessel-to-volume ratio (TVVR), hepatic vein-to-volume ratio (HVVR), and portal vein-to-volume ratio (PVVR) across three FIB-4 score categories: <1.3 (low risk), 1.3-2.67 (intermediate risk), and >2.67 (high risk) for advanced fibrosis.

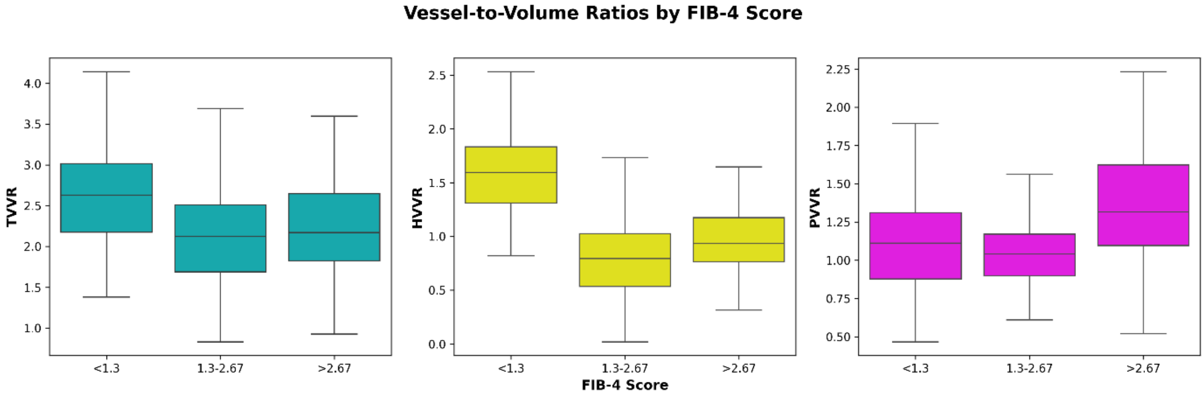

Checklist for Artificial Intelligence in Medical Imaging (CLAIM): 2024 Update

Section / Topic	No.	Item	Page / Line	No	NA
TITLE / ABSTRACT					
	**1**	Identification as a study of AI methodology, specifying the category of technology used (e.g., deep learning)	**Page 1**		
ABSTRACT					
	**2**	Summary of study design, methods, results, and conclusions	**Pages 1-2**		
INTRODUCTION					
	**3**	Scientific and/or clinical background, including the intended use and role of the AI approach	**Pages 6-7**		
	**4**	Study aims, objectives, and hypotheses	**Page 6**		
METHODS					
Study Design	**5**	Prospective or retrospective study	**Page 6**		
	**6**	Study goal	**Page 6**		
Data	**7**	Data sources	**Page 6-7**		
	**8**	Inclusion and exclusion criteria	**Page 7**		
	**9**	Data pre-processing	**Page 8**		
	**10**	Selection of data subsets	**Page 7-8**		
	**11**	De-identification methods			**x**
	**12**	How missing data were handled			**x**
	**13**	Image acquisition protocol	**Page 7**		
Reference Standard	**14**	Definition of method(s) used to obtain reference standard	**Page 7**		
	**15**	Rationale for choosing the reference standard	**Page 7**		
	**16**	Source of reference standard annotations	**Page 7**		

	**17**	Annotation of test set	**Pages 7-8**		
	**18**	Measures of inter- and intra-rater variability of features described by the annotators			**x**
Data Partitions	**19**	How data were assigned to partitions	**Page 8**		
	**20**	Level at which partitions are disjoint	**Page 8**		
Testing Data	**21**	Intended sample size			**x**

Section / Topic	No.	Item	Page / Line	No	NA
Model	**22**	Detailed description of model	**Pages 8**		
	**23**	Software libraries, frameworks, and packages	**Page 7-8**		
	**24**	Initialization of model parameters	**Page 8**		
Training	**25**	Details of training approach	**Page 8**		
	**26**	Method of selecting the final model	**Page 8**		
	**27**	Ensembling techniques			**x**
Evaluation	**28**	Metrics of model performance	**Page 8**		
	**29**	Statistical measures of significance and uncertainty	**Page 8**		
	**30**	Robustness or sensitivity analysis	**Page 10**		
	**31**	Methods for explainability or interpretability			**x**
	**32**	Evaluation on internal data	**Pages 10-11**		
	**33**	Testing on external data	**Page 10-11**		
	**34**	Clinical trial registration			**x**
RESULTS					
Data	**35**	Numbers of patients or examinations included and excluded	**Page 9**		
	**36**	Demographic and clinical characteristics of cases in each partition	**Page 9-10**		
Model performance	**37**	Performance metrics and measures of statistical uncertainty	**Pages 10-11**		
	**38**	Estimates of diagnostic performance and their precision	**Page 10-11**		
	**39**	Failure analysis of incorrect results			**x**
DISCUSSION					
	**40**	Study limitations	**Page 13-14**		

	**41**	Implications for practice, including intended use and/or clinical role	**Page 13**		
OTHER INFORMATIO N					
	**42**	Provide a reference to the full study protocol or to additional technical details			**x**
	**43**	Statement about the availability of software, trained model, and/or data	**x**		
	**44**	Sources of funding and other support; role of funders	**x**		

* Indicate page and/or line number for each checklist item that is present. NA = not applicable.
